# A Review on Antibacterial, Antiviral, and Antifungal Activity of Curcumin

**DOI:** 10.1155/2014/186864

**Published:** 2014-04-29

**Authors:** Soheil Zorofchian Moghadamtousi, Habsah Abdul Kadir, Pouya Hassandarvish, Hassan Tajik, Sazaly Abubakar, Keivan Zandi

**Affiliations:** ^1^Biomolecular Research Group, Biochemistry Program, Institute of Biological Sciences, Faculty of Science, University of Malaya, 50603 Kuala Lumpur, Malaysia; ^2^Tropical Infectious Disease Research and Education Center (TIDREC), Department of Medical Microbiology, Faculty of Medicine, University of Malaya, 50603 Kuala Lumpur, Malaysia; ^3^Department of Chemistry, Faculty of Sciences, Guilan University, Rasht, Iran; ^4^Persian Gulf Marine Biotechnology Research Center, Bushehr University of Medical Sciences, Bushehr 3631, Iran

## Abstract

*Curcuma longa* L. (Zingiberaceae family) and its polyphenolic compound curcumin have been subjected to a variety of antimicrobial investigations due to extensive traditional uses and low side effects. Antimicrobial activities for curcumin and rhizome extract of *C. longa* against different bacteria, viruses, fungi, and parasites have been reported. The promising results for antimicrobial activity of curcumin made it a good candidate to enhance the inhibitory effect of existing antimicrobial agents through synergism. Indeed, different investigations have been done to increase the antimicrobial activity of curcumin, including synthesis of different chemical derivatives to increase its water solubility as well ass cell up take of curcumin. This review aims to summarize previous antimicrobial studies of curcumin towards its application in the future studies as a natural antimicrobial agent.

## 1. Introduction

**Figure 1:**
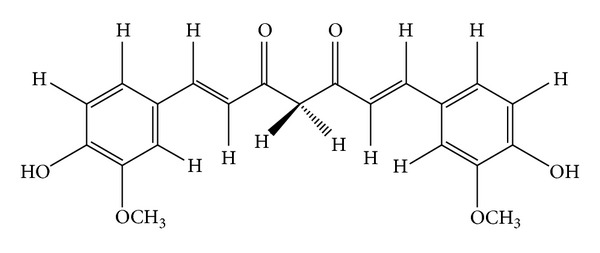
Chemical structure of curcumin.

**Figure 2:**
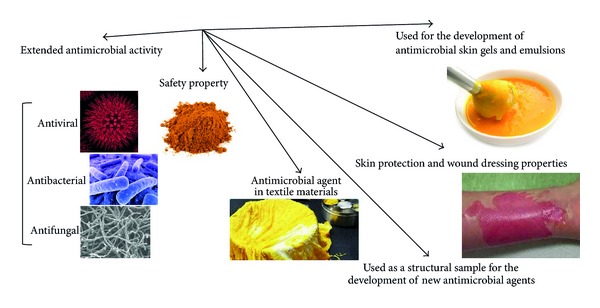
Importance of curcumin in antimicrobial studies.

Curcumin or diferuloylmethane with chemical formula of (1,7-bis(4-hydroxy-3-methoxyphenyl)-1,6-heptadiene-3,5-dione) (Figure 1) and other curcuminoids constitute the main phytochemicals of* Curcuma longa *L. (Zingiberaceae family) rhizome with the common name of turmeric [1]. This polyphenolic compound due to a variety of biological activities has been gained significant attention of researches all over the world [2–5]. Turmeric, an ancient coloring spice of Asia, as the main source of curcumin is traditionally used for many remedies [6]. As shown in Figure 2, curcumin due to a variety of specific characterizations is in interest of scientists in recent years. As many other plant materials, there are differences in the curcumin content for the* Curcuma longa* from different geographical regions and it could be due to hybridization with other* Curcuma* species which could be important fact to choose the plant with higher content of curcumin [4].

*Curcuma longa* rhizome has been traditionally used as antimicrobial agent as well as an insect repellant [7]. Several studies have reported the broad-spectrum antimicrobial activity for curcumin including antibacterial, antiviral, antifungal, and antimalarial activities. Because of the extended antimicrobial activity of curcumin and safety property even at high doses (12 g/day) assessed by clinical trials in human, it was used as a structural sample to design the new antimicrobial agents with modified and increased antimicrobial activities through the synthesis of various derivatives related to curcumin [8, 9]. It was even studied as an antimicrobial agent suitable for textile materials. Results showed that curcumin in combination with aloe vera and chitosan could be a potential suppressor for microbial growth in cotton, wool, and rabbit hair assessed by the exhaustion method [10]. Either the continuous or batch dyeing process with curcumin provided textiles with antimicrobial properties beside the color. Curcumin finished wool had semidurable antimicrobial activity, less durable to light exposure than home laundering with 45% and 30% inhibition rates against* Staphylococcus aureus* and* Escherichia coli*, respectively, after 30 cycles of home laundering [11]. Mixture of curcumin with other antimicrobial agents is used for the development of antimicrobial skin gels and emulsions with improved skin protection and wound dressing properties [12]. Composition of curcumin with hydrogel silver nanoparticles is used to increase the function of hydrogel silver nanocomposites as marked substances for antimicrobial applications and wound dressing [12]. Curcumin-loaded myristic acid microemulsion with the 0.86 *μ*g/mL of curcumin suitable for skin consumption inhibited 50% of the* S. epidermidis* growth as one of the nosocomial infectious agents. It showed 12-fold stronger inhibitory effect compared to curcumin activity dissolved in dimethyl sulfoxide (DMSO) [13].

## 2. Antibacterial Activity

Bacterial infections are among the important infectious diseases. Hence, over 50 years of extensive researches have been launched for achieving new antimicrobial medicines isolated from different sources. Despite progress in development of antibacterial agents, there are still special needs to find new antibacterial agents due to development of multidrug resistant bacteria [14]. The antibacterial study on aqueous extract of* C. longa* rhizome demonstrated the MIC (minimum inhibitory concentration) value of 4 to 16 g/L and MBC (minimum bactericidal concentration) value of 16 to 32 g/L against* S. epidermis *ATCC 12228,* Staph. aureus *ATCC 25923,* Klebsiella pneumoniae* ATCC 10031, and* E. coli* ATCC 25922 [15]. The methanol extract of turmeric revealed MIC values of 16 *μ*g/mL and 128 *μ*g/mL against* Bacillus subtilis* and* Staph. aureus*, respectively [16]. The study of hexane and ethanol turmeric extract and curcuminoids (from ethyl acetate extract of curcuminoids isolated from* C. longa* with 86.5% curcumin value) against 24 pathogenic bacteria isolated from the chicken and shrimp showed the highest antimicrobial activity for ethanol extract with the MIC value of 3.91 to 125 ppt [17]. The hexane and methanol extracts of* C. longa *demonstrated antibacterial effect against 13 bacteria, namely,* Vibrio harveyi, V. alginolyticus, V. vulnificus, V. parahaemolyticus, V. cholerae, Bacillus subtilis, B. cereus, Aeromonas hydrophila, Streptococcus agalactiae, Staph. aureus, Staph. intermedius, Staph. epidermidis, *and* Edwardsiella tarda*. However, curcuminoids elicited inhibitory activities against 8 bacteria of* Str. agalactiae, Staph. intermedius, Staph. epidermidis, Staph. aureus, A. hydrophila, B. subtilis, B. cereus, *and* Ed. tarda. *Hexane extract and curcuminoids exhibited the MIC values of 125 to 1000 ppt and 3.91 to 500 ppt, respectively [17]. Indeed, it was shown that the addition of 0.3% (w/v) of aqueous curcumin extract to the cheese caused the reduction in bacterial counts of* Salmonella typhimurium, Pseudomonas aeruginosa,* and* E. coli 0157:H7*. Moreover, it has decreased the* Staph. aureus*,* B. cereus, *and* Listeria monocytogenes *contamination after 14 days of cold storage period [18]. Turmeric oil as a byproduct from curcumin manufacture also was found effective against* B. subtilis, B. coagulans, B. cereus, Staph. aureus, E. coli, *and* P. aeruginosa *[19]. Curcumin also exhibited inhibitory activity on methicillin-resistant* Staph. aureus* strains (MRSA) with MIC value of 125–250 *μ*g/mL [20]. The* in vitro* investigation of 3 new compounds of curcumin, namely, indium curcumin, indium diacetyl curcumin, and diacetyl curcumin, against* Staph. aureus, S. epidermis, E. coli,* and* P. aeruginosa *revealed that indium curcumin had a better antibacterial effect compared to curcumin itself and it may be a good compound for further* in vivo* studies. However, diacetylcurcumin did not exhibit any antibacterial effect against tested bacteria [21]. These results demonstrated promising antibacterial activity for different curcumin derivatives as well. The stability and assembly of FtsZ protofilaments as a crucial factor for bacterial cytokinesis are introduced as a possible drug target for antibacterial agents. Curcumin suppressed the* B. subtilis* cytokinesis through induction of filamentation. It also without significantly affecting the segregation and organization of the nucleoids markedly suppressed the cytokinetic Z-ring formation in* B. subtilis *[22]. It was demonstrated that curcumin reduces the bundling of FtsZ protofilaments associated with the binding ability to FtsZ with a dissociation constant of 7.3 *μ*M. It showed that curcumin via inhibition of assembly dynamics of FtsZ in the Z-ring can possibly suppress the bacterial cell proliferation as one of the probable antibacterial mechanisms of action [22]. The study on* E. coli* and* B. subtilis* demonstrated that curcumin by the inhibitory effect against FtsZ polymerization could suppress the FtsZ assembly leading to disruption of prokaryotic cell division [23].

Also, curcumin showed significant antibacterial activity with MIC values between 5 and 50 *μ*g/mL against 65 clinical isolates of* Helicobacter pylori* [41]. Curcumin also has an inhibitory effect on NF-*κ*B activation and as a result on the release of IL-8 and cell scattering which led to a reduction in inflammation of gastric tissue as the main consequence for* H. pylori *in stomach. It inhibits the I*κ*B*α* degradation, the activity of NF-*κ*B DNA-binding and I*κ*B kinase *α* and *β* (IKK *α* and *β*) [42]. Indeed, curcumin inhibited the matrix metalloproteinase-3 and metalloproteinase-9 activity (MMP-3 and MMP-9) as inflammatory molecules involved in* H. pylori* infection in mice and in cell culture with a dose dependent manner [43]. Curcumin showed more efficient therapeutic index than conventional triple therapy of* H. pylori* on MMP-3 and MMP-9 via reduction of activator protein-1 and proinflammatory molecule activation in* H. pylori* infected gastric tissues [43].* In vivo* study of antibacterial effect of curcumin on* H. pylori* compared to OAM (Omeprazole, Amoxicillin, and Metronidazole) treatment revealed poor activity for eradication of* H. pylori* (5.9% versus 78.9% for OAM treatment). The reduction in inflammatory cytokine production was not reported from* pylori*-infected patients treated with curcumin [44]. The* in vivo* study of 1-week nonantibiotic therapy comprised of curcumin, pantoprazole, N-acetylcysteine, and lactoferrin against* H. pylori* infection was not effective for the eradication of* H. pylori*. However, the decrease in immunological criteria of gastric inflammation and dyspeptic symptoms was reported after 2 months of treatment schedule [45]. Nevertheless, the curcumin administration to the rats with* H. pylori-*induced gastric inflammation revealed a significant reduction in macromolecular leakage and NF-*κ*B activation [46]. In an* in vivo* study of* H. pylori*-infected C57BL/6 mice administered with curcumin exhibited immense therapeutic potential and pronounced eradication effect against* H. pylori* infection associated with restoration of gastric damage [41].

### 2.1. Synergistic Antimicrobial Activity

The outburst of drug resistant microbial strains necessitates the studies for synergistic effects of antibiotics in combination with plant's derivatives to develop the antimicrobial cocktail with a wider spectrum of activity and reduction of adverse side effects of antimicrobial agents.* Staph. aureus *resistance to the penicillin group of antibiotics is increasing associated with appearance of adverse side effects such as hypersensitivity and anaphylactic reactions [47]. The synergistic activity of curcuminoids and ampicillin combination demonstrated pronounced reduction in the MIC of ampicillin against either clinical strain or* Staph. aureus *ATCC 25923 strain. Bacteriocin subtilosin isolated from* B. amyloliquefaciens* in combination with encapsulated curcumin revealed partial synergism against wild-type and nisin sensitive strains of* L. monocytogenes *Scott A [48]. In another* in vivo* study using 500 *μ*g/disc of curcumin against clinical isolate of* Staph. aureus *the synergistic activity with antibiotics of cefixime, cefotaxime, vancomycin, and tetracycline was demonstrated [49]. The results proved that consumption of turmeric during the treatment of* Staph. aureus* infections with these antibiotics especially cefixime can be possibly helpful. Curcumin also demonstrated a synergistic effect in combination with some antibiotics, including ampicillin, oxacillin, and norfloxacin against methicillin-resistant* Staph. aureus *strain (MRSA) [20]. The synergistic effect of curcumin with ciprofloxacin against MRSA has also been reported, although there is an evidence of its antagonistic activity against* S. typhi* and* S. typhimurium* in combination with ciprofloxacin [49, 50].

Strongly bound metal complexes to antimicrobial agents are introduced as another possible way for synergistic activity of respective antimicrobial agents through elevation of the binding effect of them to the bacterial walls. Complexes of curcumin with cobalt nanoparticles showed increased antibacterial activity against* E. coli *[51]. Additionally, fabrication of silver nanocomposite films impregnated with curcumin showed the stronger antibacterial activity against* E. coli*. It was shown that the bactericidal activity of sodium carboxymethyl cellulose silver nanocomposite films (SCMC SNCFs) as an effective antibacterial material was improved by loading of curcumin with SCMC SNCFs [52]. In another* in situ* investigation, the synergistic effect of curcumin encapsulated chitosan-[poly (vinyl alcohol)] silver nanocomposite films was shown. The novel antimicrobial films with pronounced antimicrobial exhibition against* E. coli* proved to be potential antibacterial material for treating infections or wound dressing [53].

### 2.2. Anti-Biofilm Activity

Secretion of exopolysaccharide alginate via different stimulators such as aminoglycosides and imipenem consumption caused the increase in biofilm volume of* P. aeruginosa*. Anti-biofilm activity of curcumin against two strains of* P. aeruginosa* isolated from deep oropharyngeal swap samples of two cystic fibrosis patients with MIC values of 16 *μ*g/mL was investigated by crystal violet staining method. The curcumin treatment of the strains with MIC concentrations did not reveal noteworthy elevation in biofilm optical density [54]. In addition, in another study curcumin showed the potential for reduction of biofilm initiation genes, inhibition of 31 quorum sensing (QS) genes, and downregulation of virulence factors including acyl homoserine lactone (HSL) production, elastase/protease activity, and pyocyanin biosynthesis. The antimicrobial activities led to reduction of pathogenicity in* Arabidopsis thaliana *and* Caenorhabditis elegans *as whole plant and animal infected models with* P. aeruginosa* [7]. The results exhibited that curcumin can be a potential candidate for* P. aeruginosa* infections in special infections characterized by biofilm formation, although further comprehensive studies are needed for the approval.

In some cases the adverse effects of curcumin against different antibiotics were shown. Ciprofloxacin is the most effective antibiotic against typhoidal and nontyphoidal infection of* Salmonella.* The main mechanism for antibacterial activity of ciprofloxacin is through SOS response, induction of chromosome fragmentation, and the production of ROS in the bacterial cell. The* in vivo* and* in vitro* investigations on curcumin together with ciprofloxacin showed that, through interference with ciprofloxacin activity, it caused an elevation in proliferation of* Salmonella typhi* and* Salmonella enterica *serovar Typhimurium (*S. typhimurium*). Although curcumin could not suppress the ciprofloxacin-induced gyrase inhibition, it protected* Salmonella* against oxidative burst induced by interferon *γ* (IFN*γ*) or ciprofloxacin via owing strong antioxidant effect. The results demonstrated the curcumin by suppressing the antibacterial effect of IFN*γ* or ciprofloxacin might increase the* Salmonella* pathogenesis [55]. The study of curcumin activity in a murine model of typhoid fever exhibited an elevation of* Salmonella typhimurium* pathogenicity and increased resistance to antimicrobial agents including antimicrobial peptides, nitrogen species, and reactive oxygen. Upregulation of genes involved in antioxidative function like mntH, sitA, and sodA as well as other genes involved in resistance to antimicrobial peptides including pmrD and pmrHFIJKLM was considered as a possible cause for the mentioned elevated tolerance. Curcumin also induced upregulation effect on SPI2 genes involved in intracellular survival and downregulation activity on SPI1 genes involved for entry within epithelial cells. This information proved that the indiscriminate use of curcumin should probably inhibit the pathogenesis of* Salmonella* [55]. Additionally, curcumin also at a dose 500 *μ*g/disc showed antagonistic activity on the bactericidal effect of nalidixic acid against clinical strain of* Staph. aureus* investigated by disc diffusion method [49].

## 3. Antiviral Activity

Lack of effective therapeutics for the most of viral diseases, emergence of antiviral drug resistance, and high cost of some antiviral therapies necessitate finding new effective antiviral compounds [56, 57]. Additionally, the existing antiviral therapies are not always well-tolerated or quite effective and satisfactory [58]. Hence, the increasing requirement for antiviral substances will be more highlighted. Plants as a rich source of phytochemicals with different biological activities including antiviral activities are in interest of scientists [59, 60]. It has been demonstrated that curcumin as a plant derivative has a wide range of antiviral activity against different viruses. Inosine monophosphate dehydrogenase (IMPDH) enzyme due to rate-limiting activity in the* de novo* synthesis of guanine nucleotides is suggested as a therapeutic target for antiviral and anticancer compounds. Among the 15 different polyphenols, curcumin through inhibitory activity against IMPDH effect in either noncompetitive or competitive manner is suggested as a potent antiviral compound via this process [61]. The study of different bioconjugates of curcumin, namely, di-*O*-tryptophanylphenylalanine curcumin, di-*O*-decanoyl curcumin, di-*O*-pamitoyl curcumin, di-*O*-bis-(*γ*,*γ*)folyl curcumin, C^4^-ethyl-*O*-*γ*-folyl curcumin, and 4-*O*-ethyl-*O*-*γ*-folyl curcumin, against variety of viruses including parainfluenza virus type 3 (PIV-3), feline infectious peritonitis virus (FIPV), vesicular stomatitis virus (VSV), herpes simplex virus (HSV), flock house virus (FHV), and respiratory syncytial virus (RSV) assessed by MTT test showed the potent antiviral activity of curcumin and its bioconjugates against different viral pathogens for further studies. Also, di-*O* tryptophanylphenylalanine curcumin and di-*O*-decanoyl curcumin revealed remarkable antiviral activity against VSV and FIPV/FHV with EC_50_ values of 0.011 *μ*M and 0.029 *μ*M, respectively. However, bioconjugates did not exhibit significant antiviral activity against III_B_ and ROD strains of type 1 human immunodeficiency virus (HIV-1) in MT-4 cells [62].  Table 1 summarizes the antiviral activity of* C. longa* and curcumin and possible mechanisms underlying inhibitory effects.

Viral long terminal repeat (LTR) has a critical role in transcription of type 1 human immunodeficiency virus (HIV-1) provirus. Inhibition of LTR activity can be a possible pathway for antiviral drug candidates in order to block HIV-1 replication [63, 64]. Curcumin proved to be an effective compound to inhibit the HIV-1 LTR-directed gene expression without any major effects on cell viability [24]. Curcumin and its derivatives, namely, reduced curcumin, allyl-curcumin, and tocopheryl-curcumin, revealed 70% to 85% inhibition in Tat protein transactivation of HIV-1 LTR measured by *β*-galactosidase activities of HeLa cells which in HIV-1 LTR was fused to the indictor of* lacZ* gene. Tocopheryl-curcumin demonstrated the most inhibition activity with 70% inhibition at 1 nM compared to 35% inhibition of curcumin at this concentration [25]. In addition, curcumin inhibited the acetylation of Tat protein of HIV significantly by p300 associated with suppression of HIV-1 multiplication. Curcumin by targeting the acetyltransferase proteins of p300/CREB-binding protein (CBP) can be a potent compound for combinatorial HIV therapeutics [28]. Curcumin was found to be an inhibitor of HIV-1 and HIV-2 protease with IC_50_ of 100 *μ*M and 250 *μ*M, respectively. The curcumin boron complexes exhibited noteworthy inhibition reduced to the IC_50_ value of 6 *μ*M with time-dependent activity. The elevated affinity of boron derivatives of curcumin is possibly associated with the attachment of the orthogonal domains of the compound in intersecting sites within the substrate-binding cavity of the protease [26]. Integrase as another essential enzyme for HIV-1 replication was found to be inhibited by curcumin with IC_50_ value of 40 *μ*M. Inhibition of deletion mutant of integrase containing only amino acids 50–212 indicated that curcumin possibly interacts with catalytic core of the enzyme. The study of energy minimization and the structural analogs of curcumin elicited that an intramolecular stacking of two phenyl rings of curcumin is possibly responsible for anti-integrase activity via bringing the hydroxyl groups into close proximity [27]. However, rosmarinic acid and dicaffeoyl methane as two curcumin analogs showed noteworthy inhibitory activity against integrase of HIV-1 with IC_50_ values less than 10 *μ*M with the slow rate of binding to the enzyme assessed by kinetic studies [65]. However, through a clinical trial investigation on curcumin as an anti-HIV compound in 40 patients in eight weeks it was shown that there is no reduction in viral load or elevation in CD4 counts. But patients claimed that they preferred to take the curcumin in order to tolerate the minor gastrointestinal sufferings and feel better [29]. This demonstrated that clinical trials can possibly show up with the results completely different from* in vitro* studies. The clinical trial of clear liquid soap containing 0.5% w/v ethanol extract of* C. longa* rhizome on HIV patients reduced the wound infections and 100% decrease in itching symptom and it also affected the abscess to convert to dryness scabs (78.6%) within 2 weeks [16].

Curcumin showed the anti-influenza activity against influenza viruses PR8, H1N1, and H6N1. The results showed more than 90% reduction in virus yield in cell culture using 30 *μ*M of curcumin. The plaque reduction test elicited the approximate EC_50_ of 0.47 *μ*M for curcumin against influenza viruses [30]. In H1N1 and also H6N1 subtypes, the inhibition of haemagglutinin interaction reflected the direct effect of curcumin on infectivity of viral particles and this has proved by time of drug addiction experiment. Additionally, unlike amantadine, viruses developed no resistance to curcumin. The methoxyl derivatives of curcumin also did not show noteworthy role in the haemagglutination [30]. These results proved the significant potential of curcumin for inhibition of influenza.

*In vitro* study of curcumin and its derivatives, namely, gallium-curcumin and Cu-curcumin, exhibited remarkable antiviral activity against herpes simplex virus type 1 (HSV-1) in cell culture with IC_50_ values of 33.0 microg/mL, 13.9 microg/mL, and 23.1 microg/mL, respectively. The 50% cytotoxic concentration (CC_50_) of the respective compounds on Vero cell line showed to be 484.2 *μ*g/mL, 255.8 *μ*g/mL, and 326.6 *μ*g/mL, respectively [31]. Curcumin considerably decreased the immediate early (IE) gene expression and infectivity of HSV-1 in cell culture assays. Curcumin has an effect on recruitment of RNA polymerase II to IE gene promoters through mediation of viral transactivator protein VP16, by an independent process of p300/CBP histone acetyl transferase effect [32].* In vitro* replication of HSV-2 could be decreased by curcumin with ED_50_ value of 0.32 mg/mL [32]. Moreover, an* in vivo* study on mouse model with intravaginal HSV-2 challenge showed significant protection against HSV-2 infection due to administration of curcumin. This study showed that curcumin can be a good candidate for developing the antiviral products used intravaginally by women for protection against sexually transmitted herpes virus infection [33]. Indeed, a metallo-herbal complex of curcumin with copper (Cu^2+^) demonstrated microbicidal effect for further studies of vaginal gel with antiviral activity [66].

Coxsackieviruses cause a variety of diseases such as dilated cardiomyopathy and myocarditis. Coxsackievirus B3 (CVB3) in spite of extensive investigations is still a major human pathogen without specific effective and approved treatment [67, 68]. Curcumin exhibited the antiviral activity against coxsackievirus by reduction of viral RNA expression, protein synthesis, and virus titer. In addition, it was found to have a protective effect on cells against virus-induced apoptosis and cytopathic activity. Analysis of different pathways showed that curcumin forced its potent antiviral effect in inhibition of coxsackievirus replication through dysregulation of the ubiquitin-proteasome system (UPS) [34]. The recent studies proved that the UPS-mediated protein modification or degradation is an essential factor in the regulation of coxsackievirus replication [69].

Liver diseases associated with viral infections are major pandemics [70]. The fact that hepatitis B virus (HBV) elevates the possibility for the hepatocellular carcinoma (HCC) development some 100-fold and 695.900 deaths occurred due to liver cirrhosis and HCC worldwide in 2008 makes the need to find new antivirals against hepatitis viruses [71, 72]. The study of antiviral effect of aqueous extract of* Curcuma longa *rhizoma against HBV in HepG 2.2.15 cells containing HBV genomes showed repression of HBsAg secretion from liver cells without any cytotoxic effect. It also suppressed the HBV particles production and the rate of mRNA production of HBV on infected cells. The* Curcuma longa* extract suppressed HBV replication by increasing the rate of p53 protein through enhancing the stability of the protein as well as transactivating the transcription of p53 gene. It was understood that the extract has suppressed HBV enhancer I and X promoter leading to repression of HBx gene transcription by affecting p53 [35].* In vitro* investigation of the antiviral activity of curcumin Huh7 replicon cells expressing the hepatitis C virus (HCV) indicated that curcumin can be a potent anti-HCV compound. Results showed the decrease in HCV gene expression and replication through suppressing the Akt-SREBP-1 pathway. In addition, the mixture of curcumin and IFN*α* as the known anti-HCV therapy induced profound inhibitory activity on HCV replication and demonstrated that curcumin can be possibly used as a complementary therapy for HCV [36].

High-risk human papillomaviruses (HPVs) infection via the expression of E6 and E7 viral oncoproteins has a critical role for development of cervical carcinoma. Curcumin showed the inhibitory activity against the expression of E6 and E7 genes of HPV-16 and HPV-18 as two main highly oncogenic human papilloma viruses [37]. The transcription factor AP-1 is a critical factor for transcriptional regulation of high-risk HPVs such as HPV-16 and HPV-18. Curcumin downregulates the AP-1 binding activity in HeLa cells with decreasing effect on the transcription of HPV-18 [38]. The results showed that curcumin through apoptosis modulation and also prevention of NF*κ*B and AP-1 translocation associated with downregulation of viral oncogenes and decreasing the transcription of HPVs can be a good candidate for the management of highly oncogenic HPV infections [37, 38].

Japanese encephalitis virus (JEV) as an important endemic arbovirus in Southeast Asia is a major cause of acute encephalopathy which generally affects the children and leads to death in one third of patients. The permanent neuropsychiatric sequel is a complication for many survivors from JEV due to ineffective therapeutic measure [73]. The investigation of antiviral activity of curcumin on Neuro2a cell line infected with JEV showed reduction in production of infectious viral particles through inhibition of ubiquitin-proteasome system. The results of* in vitro* study indicated that curcumin through modulating cellular levels of stress-related proteins, reducing proapoptotic signaling molecules, restoration of cellular membrane integrity, and reduction in reactive oxygen species in cellular level imparts neuroprotection and can be a potential for further investigations [39].

Oncogenesis by human T-cell leukemia virus type 1 as an etiologic factor of adult T-cell leukemia (ATL) is critically dependent on the activation of the activator protein 1 (AP-1) [74]. The DNA binding and transcriptional effect of AP-1 in HTLV-1-infected T-cell lines were suppressed by curcumin treatment. Curcumin also inhibited the expression of JunD protein as an important factor in AP-1-DNA complex in HTLV-1-infected T-cells as well as HTLV-1 Tax-induced AP-1 transcriptional effect. Cell cycle arrest and inducing of apoptosis were found to be possible mechanisms against HTLV-1 replication in infected T-cell line by curcumin. Suppression of AP-1 activity possibly through decreasing the expression of JunD protein is introduced as a possible pathway for anti-ATL activity of curcumin [40].

## 4. Antifungal Activity

Substances and extracts isolated from different natural resources especially plants have always been a rich arsenal for controlling the fungal infections and spoilage. Due to extensive traditional use of turmeric in food products, various researches have been done in order to study the turmeric and curcumin with the aspect of controlling fungal related spoilage and fungal pathogens. The study of addition the turmeric powder in plant tissue culture showed that turmeric at the 0.8 and 1.0 g/L had appreciable inhibitory activity against fungal contaminations [75]. The methanol extract of turmeric demonstrated antifungal activity against* Cryptococcus neoformans *and* Candida albicans* with MIC values of 128 and 256 *μ*g/mL, respectively [16]. The study of hexane extract of* C. longa *at 1000 mg/L demonstrated antifungal effect against* Rhizoctonia solani*,* Phytophthora infestans, *and* Erysiphe graminis*. It was also shown that 1000 mg/L of ethyl acetate extract of* C. longa* exhibitedinhibitory effect against* R. solani*,* P. infestans, Puccinia recondita, *and* Botrytis cinerea. *Curcumin at 500 mg/L also showed antifungal activity against* R. solani*,* Pu. recondita, *and* P. infestans* [76]. Curcumin and turmeric oil exert antifungal effect against two phytophagous fungi, namely,* Fusarium solani* and* Helminthosporium oryzae*. Turmeric oil exhibited the most effective antifungal activity against* F. solani* and* H. oryzae* with IC_50_ of 19.73 and 12.7 *μ*g/mL, respectively [77]. The crude methanol extract of* C. longa *has inhibitory effect against some clinical isolates of dermatophytes. It was demonstrated that 18-month-old and freshly distilled oil isolated from rhizome of* C. longa* showed the most potent antifungal effect against 29 clinical isolates of dermatophytes with MIC values of 7.2 and 7.8 mg/mL, respectively [78].* Trichophyton rubrum*,* T. mentagrophytes*,* Epidermophyton floccosum, *and* Microsporum gypseum *were suppressed by 1 : 40–1 : 320 dilutions of turmeric oil. An* in vivo* study on infected guinea pigs with* T. rubrum* demonstrated that dermal application of turmeric oil (dilution 1 : 80) induced an improvement in healing of the lesions after 2–5 days and it caused the lesions after 6-7 days of consumption to vanish. Turmeric oil also showed activity against pathogenic molds such as* Sporothrix schenckii*,* Exophiala jeanselmei*,* Fonsecaea pedrosoi,* and* Scedosporium apiospermum* with MIC values of 114.9, 459.6, 459.6, and 114.9 *μ*g/mL, respectively [79]. However, curcumin showed more significant effect against* Paracoccidioides brasiliensis* than fluconazole, although it did not affect the growth of* Aspergillus* species [80]. The possible mechanism underlying the mentioned antifungal effect was found to be downregulation of Δ^5,6^ desaturase (ERG3) leading to significant reduction in ergosterol of fungal cell. Reduction in production of ergosterol results in accumulations of biosynthetic precursors of ergosterol which leads to cell death via generation of ROS [81]. Reduction in proteinase secretion and alteration of membrane-associated properties of ATPase activity are other possible critical factors for antifungal activity of curcumin [82].

Resistant strain development among the Candida species against existing antifungal drugs became a critical problem for therapeutic strategies. Thereby, finding new anti-Candida substances seems to be crucial [83]. The study of curcumin against 14 strains of Candida including 4 ATCC strains and 10 clinical isolates showed that curcumin is a potent fungicide compound against Candida species with MIC values range from 250 to 2000 *μ*g/mL [82]. In another study, anti-Candida activity of curcumin was demonstrated against 38 different strains of Candida including some fluconazole resistant strains and clinical isolates of* C. albicans*,* C. glabrata, C. krusei, C. tropicalis,* and* C. guilliermondii*. The MIC_90_ values for sensitive and resistant strains were 250–650 and 250–500 *μ*g/mL, respectively. Intracellular acidification via inhibition of H^+^-extrusion was identified as possible mechanism for cell death of Candida species [84]. The development of hyphae was proved to be inhibited by curcumin through targeting the global suppressor thymidine uptake 1 (TUP1) [81, 85]. Curcumin also showed inhibitory effect on* Cryptococcus neoformans *and* C. dubliniensis* with MIC value of 32 mg/L [80]. One of the major complications during therapies against chronic asthma is oropharyngeal candidiasis. Curcumin as a potential candidate for the treatment of candidosis with anti-inflammatory activity was studied in a murine model of asthma. Oral administrator of Curcumin is more effective than dexamethasone in reducing fungal burden in BALB/c mice. It also significantly decreased pathological changes in asthma [86]. Adhesion of Candida species isolated from AIDS patients to buccal epithelial cells is also markedly inhibited by curcumin and it was found to be more effective compared to fluconazole [80].

The investigation of curcumin mediation for photodynamic therapy can reduce the biofilm biomass of* C. albicans*,* C. glabrata,* and* C. tropicalis*. The results demonstrated that association of four LED fluences for light excitation with 40 *μ*M concentration of curcumin at 18 J/cm^2^ inhibited up to 85% metabolic activity of the tested Candida species. The use of curcumin with light proved to be an effective method for noteworthy improvement in the antifungal activity against planktonic form of the yeasts [87]. Photodynamic effect considerably decreased* C. albicans* viability in either planktonic or biofilm cultures probably through increasing the uptake of curcumin by cells. However, to a lesser extent, photodynamic therapy was found to be phototoxic to the macrophages. [88]. A study on a murine model of oral candidiasis was done for gathering reliable data for curcumin-mediated photodynamic therapy efficacy* in vivo*. Results proved that all exposures to curcumin with LED light markedly inhibited the* C. albicans* viability after photodynamic therapy without harming the host tissue of mice. However, 80 *μ*M of curcumin in association with light showed the best decrease in colony counts of* C. albicans *[89]. These results showed that curcumin is a high potential photosensitizer compound for fungicidal photodynamic therapy especially against Candida species.

The strong antifungal activity of* C. longa *rhizome and its low side effect were the main reasons to investigate its probable synergistic effect with existing fungicides. The synergistic activity of curcumin with five azole and two polyene drugs including voriconazole, itraconazole, ketoconazole, miconazole, fluconazole, amphotericin B, and nystatin showed 10–35-fold reduction in the MIC values of the fungicides against 21 clinical isolates of* C. albicans*. The synergistic activity of curcumin with amphotericin B and fluconazole could be associated with the accumulation of ROS which will be suppressed by adding an antioxidant [85]. The study of 200 clinical isolates of Candida species including* C. tropicalis, C. kefyr, C. krusei, C. guilliermondii, C. glabrata, C. parapsilosis, *and* C. albicans* demonstrated fungicidal activity for curcumin with MIC value of 32–128 *μ*g/mL. Combination of curcumin with amphotericin B also exhibited synergistic activity against tested Candida species, although fluconazole and curcumin in some cases showed additive effects rather than synergistic activity. These results proved that combination of curcumin with existing fungicidal agents can provide more significant effect against systemic fungal infections like candidemia and candidosis [90]. In silico analysis demonstrated that curcumin by attaching to albumin serum in a separate binding site of amphotericin B and forming the complex alleviated the adverse side effect of amphotericin B via delaying the red cell lysis. The stability and aqueous solubility of the complex of curcumin and amphotericin B with albumin serum can be a potential candidate for the treatment of visceral leishmaniasis and systemic fungal infections [91]. The* in vivo* study of combination of curcumin and piperine in murine model of Candida infection also revealed synergistic effect with noteworthy fungal load reduction in kidney of Swiss mice [85]. The mixture of curcumin and ascorbic acid against different strains of Candida also exhibited 5- to 10-fold reduction of MIC values compared to the time that curcumin was tested alone [92]. These synergistic effects showed that curcumin in combination with different fungicide materials can significantly elicit synergistic activity to enhance the efficacy of existing antifungal strategies.

## 5. Enhancing the Bioavailability and Solubility of Curcumin to Improve Antimicrobial Activities

The optimum potential of curcumin is limited because of poor oral bioavailability and insufficient solubility in aqueous solvents leading to poor absorption, fast metabolism, and quick systemic elimination [5, 93]. For overcoming this obstacle, nanocarriers like curcumin-loaded PLGA (poly lactide-co-glycolide) and curcumin nanoparticle formulation were investigated and their better bioactivity and bioavailability as well as increased cellular uptake compared to curcumin were reported [5]. Another study revealed that heat-extracted curcumin elevated the solubility of curcumin 12-fold without significant disintegration due to heat treatment. Modification of 4-hydroxy-2-nonenal (HNE) as a critical oxidation by-product involved in disease pathogenesis via cytotoxicity, genotoxicity, and mutagenicity is inhibited 80% by heat-solubilized curcumin and suggested a possible mechanism for inducing bioactivity of curcumin [94]. The study of nanocurcumin as a nanoparticle of curcumin with the size of 2–40 nm processed by a wet-milling technique, showed curcumin to be more freely dispersible in water leading to more significant antimicrobial activity against* Staph. aureus, E. coli, P. aeruginosa, B. subtilis, *and two fungi of* P. notatum *and* A. niger *due to reduced particle size and enhanced bioavailability [95, 96]. However, nanocurcumin demonstrated more noteworthy activity against Gram-positive bacteria rather than Gram-negatives [95]. In another study to improve the stability and solubility of curcumin, microencapsulation process was investigated. Microcapsule of curcumin with improved solubility is suitable as a preservative and colorant in food industry and it exhibited potent antimicrobial effect against food-borne pathogens including* E. coli*,* Staph. aureus*,* B. subtilis*,* B. cereus*,* Yersinia enterocolitica*,* Penicillium notatum, *and* Saccharomyces cerevisiae* with MIC values ranging from 15.7 to 250 *μ*g/mL. It was demonstrated that Gram-positive bacteria were more susceptible to the microcapsulated curcumin compared to Gram-negatives. However, antifungal effect was found to be stronger than the bactericidal effect [97, 98].

## 6. Conclusion

All previous investigations have shown the extensive antimicrobial activity of curcumin, although* in vivo* studies in some cases reported the less effective results of curcumin inhibitory effect. Among all former studies on antibacterial activity of curcumin the most promising result is against* Helicobacter pylori*, at least for using the curcumin as a complementary compound in combination with other existing medicines to decrease the symptoms of gastritis. The extensive antiviral effects of curcumin against different viral pathogens nominate this compound as an antiviral drug candidate to develop new antivirals from natural resources against sensitive viruses especially by developing different curcumin derivatives. However, using curcumin or its derivatives as antiviral compounds needs further investigations. Regarding the studies on antifungal activities of curcumin the most significant effect was found against Candida species and* Paracoccidioides brasiliensis, *although curcumin revealed fungicide effect against various fungi. In spite of various biological activities of curcumin, no real clinical uses have been reported for this compound and still clinical trials are undergoing for different ailments and diseases, namely, colon and pancreatic cancers, multiple myeloma, myelodysplastic syndromes, Alzheimer, and psoriasis [99]. Until 2013, more than 65 clinical trials on curcumin have been carried out, and still more is underway. This polyphenol compound is now used as a supplement in several countries, namely, China, India, Japan, Korea, South Africa, the United States, Thailand, and Turkey [100].

## Figures and Tables

**Table 1:** Antiviral activities of *Curcuma longa* L. and curcumin.

Virus	Antiviral substances	Description of antiviral activity type	Reference
HIV	Curcumin	Inhibition of HIV-1 LTR-directed gene expression	[24]
	Curcumin, reduced curcumin, allyl-curcumin, tocopheryl-curcumin	Inhibition of Tat-mediated transactivation of HIV-1 LTR	[25]
	Curcumin, curcumin boron complexes	Inhibition of HIV-1 and HIV-2 proteases	[26]
	Curcumin	Inhibition of HIV-1 Integrase	[27]
	Curcumin	Inhibition of Tat protein acetylation	[28]
	Curcumin	No antiviral effect in clinical trial	[29]
Influenza	Curcumin	Inhibition of haemagglutination	[30]
HSV-1	Curcumin, gallium-curcumin, Cu-curcumin	Reduction of HSV-1 replication	[31, 32]
HSV-2	Curcumin	Significant protection in mouse model	[33]
Coxsackievirus	Curcumin	Replication inhibition through UPS dysregulation	[34]
HBV	Aqueous extract	Suppression of HBV replication by increasing the p53 level	[35]
HCV	Curcumin	Decrease of HCV replication by suppressing the Akt-SREBP-1 pathway	[36]
HPV	Curcumin	Inhibition expression of viral oncoproteins of E6 and E7	[37]
HPV	Curcumin	Downregulation effect on the transcription of HPV-18	[38]
JEV	Curcumin	Reduction in production of infective viral particles	[39]
HTLV-1	Curcumin	Downregulation of JunD protein in HTLV-1-infected T-cell lines	[40]
